# Engaging Community-Dwelling Older Adults in Research: Qualitative Substudy of Factors Impacting Participation

**DOI:** 10.2196/74191

**Published:** 2025-06-03

**Authors:** Bryah Boutilier, Grace Warner, Brianna Wolfe, Sorayya Askari, Elaine Moody, Parisa Ghanouni, Tanya Packer

**Affiliations:** 1 School of Health Administration Dalhousie University Halifax, NS Canada; 2 School of Occupational Therapy Dalhousie University Halifax, NS Canada; 3 School of Nursing Dalhousie University Halifax, NS Canada; 4 Department of Nursing Umeå University Umea Sweden

**Keywords:** older adult, aging in place, research participation, community-based research, engagement

## Abstract

**Background:**

Innovative approaches to community-level data collection are crucial to inform policies and programs that support people in aging well within their communities. For example, community-level data can proactively identify unmet health needs, inform preventative care strategies, and ensure the equitable distribution of resources that enable older adults to age in place.

**Objective:**

This paper presented a substudy of a larger community-based project designed to identify community-dwelling older adults’ concerns about their well-being and connect them with resources to help them age well at home. The substudy aimed to identify motivations that influence older adults’ engagement in research and barriers to their participation.

**Methods:**

Data collection involved qualitative semistructured interviews with 27 older adults, with a mean age of 77 (SD 5.4), who had completed a comprehensive assessment. Purposeful sampling prioritized older adults who lived in rural areas, had more than one health condition, and represented diverse ethnicities, while attempting to reach equal numbers of participants across the participating communities. Interviews were conducted by trained research team members using an interview guide focused on reasons for research participation and perceptions of the assessment and resource action plan. Meeting minutes, gathered during 35 biweekly or monthly sessions with community coordinators, captured real-time reflections on recruitment processes, challenges, and community-specific factors influencing participation. Thematic analysis was completed using both inductive and deductive approaches.

**Results:**

Older adult participants were primarily female (n=22, 82%), of European (n=19, 70%) or Acadian (n=8, 30%) descent, university educated (n=14, 52%), with one or more chronic health conditions (n=26, 96%). Older adults reported 2 main reasons for participating: planning for the future and helping their community. At the same time, barriers to participation identified included communication challenges, fear of scams, and institutional skepticism. Participants emphasized a desire for practical outcomes from the research, especially related to aging-in-place supports. Although trust in local, personal relationships facilitated participation, skepticism toward institutions and digital communication channels were barriers to participation.

**Conclusions:**

This research highlighted the need to tailor communication strategies to older adults by understanding factors influencing engagement. Addressing institutional skepticism and leveraging trusted community members are possible strategies to overcome barriers to successful engagement in community-based research. These findings advance our understanding of why older adults participate in research and suggest ways to improve recruitment strategies. Participation was motivated not only by personal benefit but also by a strong sense of civic responsibility, social connection, and a desire to contribute to future community well-being. Framing research as community-driven and future-oriented, rather than problem- or deficit-based, studies can resonate more deeply with older adults. Integrating research within existing, trusted local networks and venues helps build legitimacy and accessibility—especially in rural contexts where institutional trust may be low and digital communication less effective.

## Introduction

### Background

Understanding the health concerns among older adults living in the community is vital for adequate, preventative health care planning, resource provision, and allocation [1]. Because much of the existing data on health issues among older adults are derived from hospital and institutional settings, it is imperative to gather community-level data to inform policies and programs that support aging well [2-4]. Significant barriers [5,6] exist to obtaining comprehensive community-level data [7]. Engaging older adults in research is one solution to identify resource needs to support older adults to age in place [8].

Understanding what influences older adults to participate in community-level research and contribute data is critical to research best practices [7,9]. Barriers to participation previously identified include trust, confidentiality concerns, and communication [5,6]. Overcoming these barriers is essential to collect meaningful community-level data. Motivations that drive older adults to engage in research have also been identified. Caring about the outcomes of the study, personal characteristics (ie, curiosity, civic engagement, interest in the topic), and altruistic benefits appear to influence participation [1].

This paper presented a qualitative substudy that emerged from the *ACTing Collectively* project. The main study assessed the feasibility and utility of an innovative tool that gathers data on the needs of older adults living in the community and matches these to local resources. The substudy analysis of gathered qualitative data examined the research question “What motivates or hinders older adults’ participation in community-based health research?”

### Study Context: The ACTing Collectively Project

The overall study, *ACTing Collectively*, was carried out in Nova Scotia, Canada, with recruitment in 3 provincial municipalities, which were selected through a competitive expression of interest process involving 3 stages. After attending a webinar, interested municipalities were selected based on the following inclusion criteria: (1) a completed expression-of-interest form (ie, containing information about demographic characteristics of older adults in the municipality, the level of social and material deprivation, and the regional use of medical care), (2) an identified individual who would serve as a community coordinator to help with participant recruitment and participate in regular team meetings, and (3) willingness and capacity to act on recommendations based on project results. Submitted expression-of-interest forms allowed researchers to assess the municipal capacity and select municipalities with higher provincial deprivation and medical use levels. The following sections outline information specific to the methodology used in the *ACTing Collectively* project.

The *ACTing Collectively* project tested the feasibility of the Age Care Technologies Assess & Connect (ACT) tool and process to collect community-level data and match available resources to the specific needs of each older adult. The ACT assessment tool consists of 56 questions that assess well-being, independence, social engagement, and health, as well as a customizable resource database that can connect relevant resources to each of the 56 questions. A resource action plan is then generated and given to the older adult, listing their prioritized concerns and selected resources.

Various recruitment strategies were used to raise awareness about the *ACTing Collectively* project. These included infographics and flyers posted at different community locations (eg, health centers, churches, seniors’ clubs, libraries, and municipal offices). Printed materials outlined the research objectives, participation requirements, benefits, and project contact information (ie, phone number and email). Newspaper articles, radio/television interviews, newsletters, and posts on social media platforms supplemented this. To build relationships with the participating municipalities, the research team travelled to research sites to present the project at local events aimed at older adults. Community coordinators also continually promoted the project and recruited older adults by delivering presentations at seniors’ events. Recruitment messaging was informed by the community coordinators and included wording designed to motivate older adults (eg, participation may help your friends or other struggling older adults in the community, it may help you in the future, or it may connect you with resources).

Inclusion criteria for the ACT assessment were (1) being a community-dwelling older adult aged 65 years or more, (2) living in 1 of the 3 collaborating municipalities, and (3) being able to speak and understand English or French. Individuals who could not read/write English or French were eligible, given that a trained assessor conducted all assessments in person, virtually, or over the phone. Additionally, family caregivers (unpaid) for older adults who met the inclusion criteria were invited to participate on behalf of the older adults if barriers to participation existed (eg, health condition, hearing impairment). The single exclusion criterion was severe cognitive or psychological impairment that would hinder participants’ understanding of the assessment questions, assessed by their ability to follow instructions.

Older adults interested in participating contacted the research team to obtain information, establish eligibility, and provide informed, voluntary consent. If the family caregiver was participating on behalf of the older adult, consent was received from the caregiver and the older adult. Each participant was assigned a participant ID to protect confidentiality. The ID was used in all subsequent data collection, and the decoder was kept in a password-protected file.

Participants chose virtual, phone, or in-person interviews. The interviews were conducted by trained assessors who signed confidentiality agreements and received procedural training.

## Methods

### Substudy: Understanding the Barriers to and Facilitators of Research Involvement

This paper reported findings from (1) follow-up interviews with a sample of people who participated in the ACT assessment and (2) minutes and research study documents to better understand the barriers to and facilitators of participation in a community research project. Using a qualitative design, the research questions were as follows:

- Why do older adults choose to participate in community-based research?
- Why do older adults choose not to participate in community-based research?

### Sampling and Data Collection for the Qualitative Substudy

A subsample of older adults who completed the ACT assessment and provided consent to be recontacted were contacted by the research team 2-4 months after the ACT assessment to reconfirm their interest in participating in a follow-up interview and verbally complete the substudy informed consent process. Purposeful sampling prioritized older adults who lived in rural areas, had more than one health condition affecting their everyday life, and represented diverse ethnicities, while attempting to reach equal numbers of participants across the 3 municipalities. Five rounds of sampling were conducted throughout the study.

Those who agreed to be interviewed participated in an approximately 1-hour interview conducted over the phone by a research team member trained in qualitative interviewing techniques, such as probing. The interview was designed to determine the usefulness and feasibility of the ACT assessment and resource action plan. Areas of inquiry included descriptions of their living situation, experience completing the ACT assessment, applicability of the questions to their well-being, reasons for participation in the research, facilitators of and barriers to accessing resources identified in their resource action plan, and opinions on the usefulness of the resource action plan. This substudy concentrated on the responses to the questions regarding older adults’ reasons for participating in the study.

In addition to interviewing older adults, meeting minutes with community coordinators and other research partners were analyzed. The project’s local community coordinators were primarily responsible for on-the-ground participant recruitment and often completed the assessment. Given that data could not be gathered from those who did not agree to participate in the parent project, information from the community coordinators was key to identifying possible barriers to participation. During the 2 years of the project, there were biweekly or monthly meetings (N=35 in total) to discuss and monitor the progress and challenges in each municipality. Meeting minutes captured discussions on recruitment efforts, barriers, and new recruitment strategies. The minutes provided an opportunity to understand the community coordinators’ perspectives on why older adults chose to or did not participate in the project. Community coordinators agreed and consented to the use of minutes in data collection. All meeting minutes were used in the analysis.

### Analysis

Any identifying information was removed during the transcribing process. Pseudonyms were created for older adult participants using popular names for individuals aged over 65 years. Meetings were audio-recorded and written minutes produced by a research team member. Meeting minutes were sent to all attendees, including community coordinators, to ensure accuracy before finalizing the minutes. Any errors noted were corrected. Pseudonyms were not assigned to community coordinators due to the small sample size and risk of identification. Instead, quotes taken from minutes were not attributed.

The interviews and meeting minutes were uploaded into NVivo12 for Mac qualitative software for analysis (Lumivero). A codebook was created using a combined inductive and deductive approach. The deductive framework was based on questions in the interview guide, while the inductive approach allowed unexpected findings to arise. Combining inductive and deductive approaches recognizes existing theoretical knowledge, while also allowing new insights to emerge from the data [10]. The same codebook was used to analyze interviews and meeting minutes focused on strategies to engage older adults, ways to increase participation, and challenges experienced. Qualitative thematic analysis was used to organize the codes into patterns that evolved into themes [11]. Next, through an iterative review, themes were defined, named, and validated to represent the data accurately. Older adults’ interviews and meeting minute data were analyzed separately, and themes were compared and integrated. Member checking and intercoder reliability processes were implemented to ensure trustworthiness was enacted and the data were collected, analyzed, and interpreted accurately.

### Reflexivity

As researchers, we acknowledge that our personal characteristics, professional backgrounds, and prior experiences have the potential to shape all stages of the research process. The authors brought diverse professional backgrounds to the study, including occupational therapy, health administration, nursing, public policy, and health promotion. The research team comprised both senior researchers with extensive experience and newer research staff. We recognize that our attributes, such as all authors being female, may have influenced our interactions with participants and the interpretation of findings. Efforts were made to remain reflexive throughout the research process by meeting regularly to minimize bias and enhance the credibility and transferability of the results.

### Ethical Considerations

This research was approved by the Nova Scotia Health Research Ethics Board (ethics approval number: 1027295) and followed all requirements to protect participants’ privacy and confidentiality. All participants and community coordinators provided written or verbal informed consent before participation. Demographic data were collected via data entry by assessors. No personal identifiers were entered. Participant contact details, used to make appointments, were kept separate from the data. Interview data were deidentified during transcription and prior to analysis. Identifiable personal information was kept in a secure drive, and findings were reported in a way that ensured anonymity. Participants were entered into a CAD $100 gift card draw and received a local resource package (ie, resource action plan) in exchange for participation. No images or identifiable information were used in this manuscript.

## Results

### Participant Details

Of the older adults who completed the ACT assessment, 186 (84%) also agreed to be contacted by phone to participate in a follow-up interview. Using maximum variation sampling, 44 (24%) participants were contacted, and 27 (61%) of those contacted agreed to participate. Participants who declined to participate indicated they were too busy or no longer interested. The sample had a mean age of 77 years, and based on postal codes, [12] 48% (n=13) lived in rural areas. Participants were able to choose more than one ethnicity. They were predominantly of European (n=19, 70%) or Acadian descent (n=8, 30%), aligning with the local population’s demographic data for this age range [13]. The majority of older adult participants were female (n=22, 82%), and approximately 52% (n=14) reported some level of university education. Almost all participants reported having at least one chronic health condition (n=26, 96%). Demographic information for community coordinators was not provided, given the small sample size (n=3). Although participants varied in gender, location, and ancestry, we did not purposively sample according to these characteristics, and they did not emerge as strong themes in our analysis. In addition, limited gender variation in our larger parent study restricted our ability to purposively sample men for this substudy, thereby limiting analysis of thematic trends across gender.

### Themes and Subthemes

**Figure 1:**
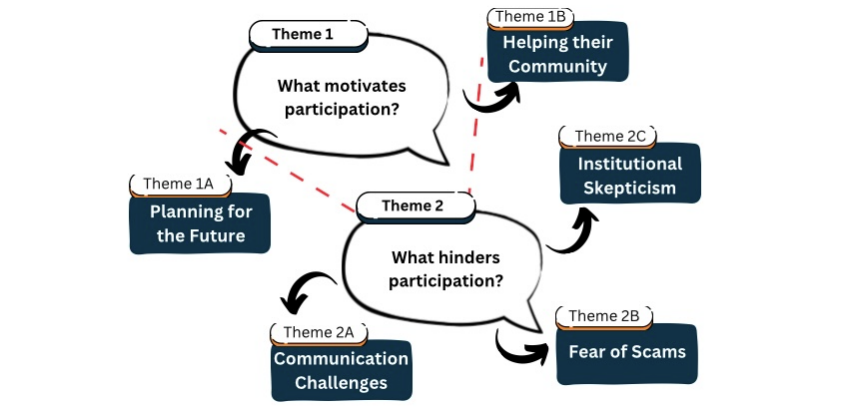
Thematic map of themes and subthemes exploring factors influencing older adults’ (65 years and older) participation in community-based research.

Two main themes emerged to explain what motivates or hinders older adults’ participation in research (Figure 1) and were organized into subthemes: theme 1 (What motivates participation?), with subthemes 1A (*planning for the future*) and 1B (*helping their community*), and theme 2 (What hinders participation?), with subthemes 2A (*communication challenges*), 2B (*fear of scams*), and 2C (*institutional skepticism*).

#### Theme 1: What Motivates Participation?

Older adults’ interviews informed theme 1. Many participants expressed that their primary motivations for engaging in the research were personal concerns, such as planning for their future or contributing to the well-being of their community.

##### Subtheme 1A: Planning for the Future

“Planning for the future” was a dominant theme. Topics such as financial planning, home maintenance or improvement, access to helpful resources (ie, services, programs), and social support systems were areas of particular interest. Participants demonstrated a proactive approach to future planning by drawing a direct link between participation in the research and understanding available resources for future needs. They shared their concerns about aging as the reason for finding out what resources are available, allowing them to plan for the future. The following quotes illustrate participants’ acknowledgment of future needs and the importance of learning how to put plans into place for the future.

I think the reason I put myself forward for this was to just find out what’s available for seniors, like if you needed help . . .

Trina

That’s exactly why we got into this sort of study that you’re doing . . . We figured one of these days we may need something to help out, and if we have some of this information, we’ll know who to call.

Thomas

A lot of it is learning about, you know all the issues that come up . . . it’s not too long in the future either that could be problematic for us . . . and you want to make sure that things get put into place before because we are an aging population.

Margaret

In addition to a general desire to plan for the future, motivation for many older adult participants was driven by specific concerns about future health or mobility challenges and the need for home modification. When participants described strategies to remain in their homes longer, they spoke of maintenance, upkeep, and accessibility services. Participants articulated the challenges older adults face in maintaining their homes, reflecting on the need for supportive or informal services, such as lawn services from a neighbor. Some older adult participants had already experienced poor health, preventing them from caring for their homes. These experiences were at the forefront of their minds and added to the urgency of identifying support to remain at home.

Last year after surgery, I had to get somebody to do the lawn . . . I’m on like 4 acres of land here . . . But I don’t know how long I can [keep maintaining land] . . . I also was just sent some scans, and I have spinal stenosis . . . you know, it’s not too bad right now, so hopefully, that doesn’t become a big issue with me because that could have a bearing on whether I can stay in my home.

Trina

##### Subtheme 1B: Helping Their Community

Many older adult participants were motivated by a sense of civic responsibility and a desire to effect positive change in their communities. They emphasized the importance of active involvement in research, seeing it as a proactive way to address community issues.

We have to speak out on behalf of our senior citizens or our people in the community. We can’t just sit back and be armchair enthusiasts and say nothing but complain about it all the time.

Anne

Altruistic motivations were prevalent among participants, as many desired research findings to benefit others in their community. Many acknowledged that the research experience did not necessarily have a direct personal impact on them, but they hoped that information could benefit others.

The whole experience with [assessor’s name] has not done anything for me personally, but I’m hoping that with the information you get, it may help other people.

William

A perceived neglect of older adults within the community fueled many to participate. OOne older adult (Helen) participated “just because the word ‘senior’ was in it . . . because it [the research] talked about seniors, and we’re often a forgotten group.

Participants expressed a perception that not enough is being done to improve the well-being of older adults in their community, underscoring the importance of amplifying the voices of older adults by incorporating their perspectives into community initiatives.

I think checking in with any older person gives them a voice that they don’t have in the ordinary community life.

Elizabeth

The following quote highlights the sense of neglect among older adults, prompting a desire to be heard and considered in community initiatives such as research:

And then they say, well, they’re doing this for seniors and that for seniors. Well, forget it because people like us [seniors] are thrown to the curb. That’s why I did it [participated in the research].

Catherine

Older adult participants expressed optimism that the research would be able to understand and address the aging population’s needs, hoping for positive changes in the well-being of older adults in their community. Participants shared their positive feelings about the research, with one participant (Dorothy) saying, “I’m pleased to know that this program is put together and that at least trying to find out what can be done for the aging population . . . When you age you just want to be able to live well.”

#### Theme 2: What Hinders Participation?

Both older adult interviews and meeting minutes informed theme 2. Three subthemes were identified: *communication challenges*, *fear of scams*, and *institutional skepticism.*

##### Subtheme 2A: Communication Challenges

As noted by community coordinators, rural communities face the dual challenge of geographic dispersion and reduced availability of print newspapers, prompting a reliance on digital publications that may bypass older adults who are less familiar with the technology. This was echoed by older adult participants who preferred receiving information through traditional means, such as print or phone communication. One older adult participant (Ruth) said, “I’d say there’s a lot of older people that are not with the computers and that. They just need to get it [hear about research] on paper or phone because they can’t go online.”

Effectively disseminating information to older adults, with limited access to traditional communication channels, such as newspapers, challenged project recruitment. One community coordinator stated, “I anticipate that our greatest challenge will be to educate the public about the project and recruit enough participants to be assessed!”

##### Subtheme 2B: Fear of Scams

A fear of falling victim to fraudulent activities hindered the willingness to engage in research. Despite the research team’s efforts to reassure participants through clear communication protocols, legitimate concerns about scams remained. One community coordinator stated, “There is a lot of fear in communities right now about fraud and scams, and I do think that is probably having an impact on recruitment.”

Older adult participants also shared their hesitancies with phone and email technology because of scams:

The only thing I think of now are so many scams that are out there . . . like I got scammed once or twice before I even knew anything about a scammer.[Ruth]

Calling older adult participants and leaving voicemails multiple times, naming and identifying relationships with the project and always telling participants when to expect a call-back appeared to mitigate this fear but only to a limited extent. Older adult participants agreed that this was useful in making them feel more secure.

I think it was good the way you called ahead of time and set an appointment time. Just there’s so many scammers out there now, so it’s good to know that, okay, you’re going to call somewhere around 11 o’clock on such and such a day . . . So many elderly people don’t realize when somebody calls [that it could be a scam].

Helen

##### Subtheme 2C: Institutional Skepticism

Broader societal attitudes toward institutional entities, including skepticism toward government-funded initiatives and research endeavors, were also reported as barriers to participation. A community coordinator spoke of resentment in their communities for things that seemed to be government-related, including research:

What I hadn’t anticipated was the level of resentment from the public toward the government and large bodies and research . . .

Participants’ reluctance to engage with government-funded navigation services, such as toll-free information phone lines, reflected a more profound mistrust of bureaucratic institutions and a preference for local community-based support networks.

I’m going to tell you right now; I don’t have to call them [government navigation agencies] when I know that they’re going to be useless . . . Anything to do with the government, you’re going to get a run-around.

Martha

This skepticism was also reflected in attitudes toward publicly funded research. A lack of engagement, even at in-person events, was interpreted by community coordinators as further proof of public skepticism:

There is resentment about pouring resources into ”research” instead of simply meeting basic needs.

I was able to offer a presentation about the ACTing Collectively project . . . It was surprising that even though I put the sign-up sheets that [the research team] sent yesterday on each table and asked everyone present to consider adding their name to indicate that they agree to be contacted to consider participating, only 4 of about 50 people provided their contact information.

Leveraging personal relationships and trusted community champions was observed to overcome or mitigate this institutional skepticism. Community coordinators shared their thoughts:

Seeing the research team relax during the [older adult event] visit was valuable . . . It was nice to make the personal connections with them and the seniors.

However, community coordinators still felt that “recruiting is best done by someone in the community . . . someone who is trusted,” emphasizing the importance of involving local partners.

## Discussion

### Principal Findings

This study explored the factors that influence older adults’ participation in research and the barriers that hinder such engagement. Older adults were motivated to participate in planning for the future and helping their community.

#### Motivations for Participation

##### Proactive Approaches

Participants in this study echoed what is known about planning for the future, linking this to their decision to participate in the research. A fear of loss of autonomy, independence, and frailty underpin older adults’ need to plan for the future [14-16]. A proactive approach is positively associated with dimensions of successful aging, such as the ability to care for oneself and adjust to age-related changes [17]. Conversely, the literature also states that higher rates of depression in older adult populations are associated with avoidance of planning for the future [18,19]. Although most participants in this study had one or more chronic health conditions, the sample was relatively healthy and educated, aligning with known research about populations that engage in research [20].

Concerns about mobility challenges and the need for home modification emerged as motivations for participation. Research highlights the interconnection between health challenges during the aging process and the need for home maintenance [21]. Additionally, participants in this study recognized the importance of socialization for overall well-being, including support for others in their community. This may have underpinned their motivation to plan for the future, as it echoes the existing research that social isolation is negatively associated with positive mental health, successful aging [22], and quality of life in older populations [14,19].

##### Civic Responsibility

Participants’ motivations reflected a sense of civic responsibility and a commitment to effecting positive change within their community. Similar to our findings, previous studies examining volunteerism among older adults have found altruism to be a primary motivator for engagement in activities that may benefit the community [1]. Active engagement in research was perceived as a means to address community issues proactively, aligning with previous studies emphasizing the role of community engagement in promoting well-being among older adults [20,23,24].

Participants in this study expressed concerns about the perceived neglect of older adults within the community, highlighting the importance of incorporating their perspectives into community initiatives. Studies have shown that older adults often feel marginalized or overlooked in society, leading to a desire for greater inclusion in the community and recognition of their needs [19,25]. The existing literature emphasizes the need to amplify the voices of older adults in shaping policies and interventions to address their unique needs [26,27], which may motivate older adults to believe in their ability to shape their community.

#### Barriers to Participation

The literature highlights the importance of using multiple communication channels to reach diverse populations, particularly those with limited digital literacy [28]. Even though print newspapers are less available in rural areas, this study found that published newspapers are a preferred recruitment method. Additionally, the move to digital publications makes it even harder for older adults who are not tech-savvy to stay informed. Strategies such as personalized outreach and community-based intermediaries may help bridge this communication gap [29].

In this study, older adults were skeptical about scams and adopted behaviors to protect themselves, such as not answering phone calls and confirming caller identification. Research indicates that older adults are particularly vulnerable to scams and fraud due to social isolation and cognitive decline [30]. Using transparent and secure recruitment methods, including providing educational resources on recognizing and avoiding scams, may help alleviate these concerns and enhance trust in research initiatives [31].

Skepticism toward institutional entities, particularly government-related initiatives, presented a challenge to research recruitment efforts in this study. In rural communities, skepticism may be amplified due to a history of perceived neglect of resources and health care services [32]. Deep-seated mistrust of bureaucratic institutions and a preference for local community-based supports hinder engagement with research because it is perceived as associated with the government. This finding underscores the importance of building personal relationships and leveraging trusted community members as recruitment champions to overcome institutional skepticism. Engaging with local partners and involving community members in the research process can foster trust and legitimacy, ultimately enhancing participation [33,34].

### Implications for Researchers

This study underscored the importance of aligning research approaches with participants’ motivations, values, and communication preferences. Participation was notably influenced by how the parent study was framed. Emphasizing local needs, future planning, and community benefit resonated deeply with older adults’ desire to help others, give back to their community, and have their voices heard. This community-oriented framing—rather than a focus on personal diagnosis or treatment—had positive benefits that may have reduced stigma and increased comfort with participation. Recruitment strategies that leverage relationships and trust held by local partners (eg, Seniors’ Safety Program coordinators, community health workers) can play a critical role in overcoming institutional skepticism. These trusted intermediaries help validate the project’s legitimacy and make initial contact more approachable.

Additionally, using physical recruitment materials in familiar, high-traffic community locations (eg, post offices or pharmacies) and offering brief, in-person researcher outreach during existing gatherings (eg, church events, library talks), provide accessible and trusted touchpoints. To further reduce concerns about scams—a recurring barrier in this study—teams should also consider providing verification tools, such as local phone numbers or letters of affiliation from known organizations.

Together, these insights suggest that the way research is framed and embedded within community relationships can meaningfully influence older adults’ participation. By tailoring strategies to reflect older adults’ values and preferred modes of engagement, researchers can foster greater inclusion, trust, and long-term partnership in community-based aging studies.

### Implications for Policy

This research highlights several implications for policy, particularly in relation to aging populations. Participants’ strong interest in planning for the future identifies the need for communities and organizations to develop and tailor resources that are proactive and prevention focused, rather than reactive. Policies should support the design and implementation of programs that help older adults maintain independence and well-being before significant health or social challenges arise. In addition, the findings emphasize the value of delivering services at the local or community level. Older adults often prefer to engage with familiar individuals and trusted organizations, suggesting that investment in community-based service infrastructure can enhance accessibility, trust, and uptake.

### Limitations

A key limitation of this study is that the data were collected from individuals who ultimately chose to participate in the research. As such, their insights into participation barriers may not have fully captured the perspectives or motivations of those who declined to participate, potentially limiting the generalizability of findings related to participation hesitancy. However, the inclusion of data from the community coordinators partially mitigated this limitation. Additionally, since the study sample was relatively healthy and well educated, motivating factors and barriers hindering participation reflect older adults who participated. This may have led to underestimating barriers or overestimating motivations to participate on behalf of their future self or community. If a more vulnerable sample of older adults had been recruited, there may have been more individuals who were seeking resources for themselves or identified additional accessibility barriers. Lastly, this study recruited a limited number of males. We explored the possibility of identifying thematic trends across gender, location, and ancestry; however, we had not purposively sampled these characteristics, nor did they emerge as strong themes in our analysis. To explore these differences, future studies should consider sampling strategies that allow for this type of analysis.

Future research should develop and test methods to ethically engage vulnerable older adults, such as those of a lower socioeconomic status, with less education, and with more chronic health conditions. Additionally, looking at the factors impacting engagement with research among diverse ethnicities and cultures would be informative to better reach marginalized populations and broaden perspectives on findings.

### Conclusion

In summary, recognizing the factors influencing research engagement among older adults informs future studies on how they may maximize the recruitment of older adults. Highlighting the proactive involvement and altruistic motivations of older adults in research emphasizes the importance of incorporating their perspectives into study design. By comprehensively understanding the concerns and viewpoints of older adults, researchers can devise tailored recruitment strategies, interventions, and support mechanisms for research. Moreover, addressing communication barriers, apprehensions regarding scams, and institutional distrust is essential in fostering relationships to increase research participation among older adults in rural areas. Researchers can improve accessibility for older adult populations by adopting inclusive communication methods and implementing safeguards against fraudulent activities. Additionally, establishing community trust through grassroots involvement is essential. Future research can use these insights to increase the engagement of older adults in community-based research, thereby enriching scientific knowledge and the well-being of this demographic.

## Abbreviations

ACT: Age Care Technologies Assess & Connect

## Data Availability

The datasets generated and analyzed during this study are not publicly available in order to maintain the confidentiality of participants and uphold the study’s agreement with funding partners.
